# The favorable role of homozygosity for killer immunoglobulin-like receptor (KIR) A haplotype in patients with advanced-stage classic Hodgkin lymphoma

**DOI:** 10.1186/s13045-016-0255-4

**Published:** 2016-03-16

**Authors:** Giorgio La Nasa, Marianna Greco, Roberto Littera, Sara Oppi, Ivana Celeghini, Rossella Caria, Sara Lai, Rita Porcella, Massimo Martino, Alessandra Romano, Francesco Di Raimondo, Andrea Gallamini, Carlo Carcassi, Giovanni Caocci

**Affiliations:** Hematology Unit, Department of Medical Sciences “M. Aresu”, University of Cagliari, Via Is Guadazzonis, 3, 09126 Cagliari, Italy; Bone Marrow Transplant Center, R. Binaghi Hospital, ASL 8, Cagliari, Italy; Regional Transplant Center, R. Binaghi Hospital, ASL 8, Cagliari, Italy; Hematology Department and BMT Unit, Azienda Ospedaliera S. Croce e Carle, Cuneo, Italy; Hematology and Bone Marrow Transplant Unit, Azienda Ospedaliera BMM, Reggio Calabria, Italy; Division of Hematology, Azienda Policlinico-OVE, University of Catania, Catania, Italy; Research, Innovation and Statistics Department, Centre Antoine Lacassagne, Nice Cedex 2, France; Medical Genetics, Department of Medical Sciences “M. Aresu”, University of Cagliari, Cagliari, Italy

**Keywords:** Hodgkin lymphoma, Killer immunoglobulin-like receptors, KIR A haplotype, Natural killer cells, Positron emission tomography

## Abstract

**Background:**

Interim positron emission tomography after 2 cycles of ABVD (iPET-2) is a good predictor of outcome in advanced-stage classic Hodgkin lymphoma. So far, there are no other prognostic biomarkers capable of identifying chemotherapy refractory patients with comparable accuracy. Despite the considerable amount of evidence suggesting that antitumor immune surveillance is downregulated in classic Hodgkin lymphoma (cHL), few data exist on the impairment of natural killer cell function and the role of their killer immunoglobulin-like receptors (KIRs).

**Methods:**

We investigated KIR gene frequencies, KIR haplotypes, and KIR-ligand combinations in a cohort of 135 patients with advanced-stage classic Hodgkin lymphoma and 221 healthy controls. We furthermore evaluated the correlation of KIR genes and KIR haplotypes with the achievement of negative iPET-2.

**Results:**

In the cohort of patients, the 5-year overall survival and progression-free survival were 93.6 and 79 %, respectively. Homozygosity for KIR A haplotype and the HLA-C1 KIR ligand (KIR-AA/C1C1) was significantly higher in healthy controls (15.7 vs. 4.8 %, *p* = 0.001). The KIR-AA genotype resulted to have a significant predictive power for achieving iPET-2 negativity (*p* = 0.039).

**Conclusions:**

Homozygosity for KIR A haplotype offers protection against classic Hodgkin lymphoma. The association found for the KIR-AA genotype and achievement of negative iPET-2 suggests that KIR-AA could be used in clinical practice to enhance the chemosensitivity predictive power of iPET-2. Our results point to the possibility of adapting treatment strategies based on the combination of KIR biomarkers and PET scan.

## Background

Cure rates in classic Hodgkin lymphoma (cHL) range from 70 to 90 %, but complete recovery of patients is burdened by long-term toxicities of chemoradiation treatment, particularly heart and lung injury and secondary neoplasms [1]. Although in recent years interim [^18^F]-fluoro-2-deoxy-d-glucose positron emission tomography carried out after 2 cycles of doxorubicin, bleomycin, vinblastine, dacarbazine (ABVD) treatment (iPET-2) has emerged as a powerful predictor of treatment outcome in advanced-stage cHL patients [2], no other biomarkers have been shown capable of predicting treatment outcome at baseline with the same accuracy. Because of its unique histology, cHL is an interesting study model for the identification of new immunological and immunogenetic factors that may confer susceptibility to tumor or influence response to treatment [3]. In fact, the peculiar architecture of “malignant lymphogranuloma” is characterized by the presence of only a few neoplastic cells—Hodgkin-Reed-Sternberg (HRS) cells—growing in a microenvironment rich with immune system cells that are incapable of mounting an effective antitumor response [4].

Despite the considerable amount of evidence suggesting that antitumor immune surveillance is downregulated in cHL [5], few data exist on the impairment of natural killer (NK) cell function in cHL. NK cells are a key component of the innate immune system and play an important role in antitumor surveillance. Their activity is regulated by several receptor families, including killer cell immunoglobulin-like receptors (KIRs) which recognize human leukocyte antigen (HLA) class I molecules expressed on target cells [6]. NK cells express different combinations of the *KIR2DL1*, *KIR2DL2*, *KIR2DL3*, *KIR2DL4*, *KIR2DL5*, *KIR3DL1*, *KIR3DL2*, and *KIR3DL3* inhibitory KIR genes and *KIR2DS1*, *KIR2DS2*, *KIR2DS3*, *KIR2DS4*, *KIR2DS5*, and *KIR3DS1* activating KIR genes [7]. The KIR gene family has been divided into two broad groups of haplotypes according to gene content. KIR A haplotypes encode the *KIR2DL3*, *KIR2DL1*, *KIR3DL1*, and *KIR3DL2* inhibitory genes and the *KIR2DS4* activating gene. These haplotypes have identical KIR gene content, but can differ for allelic polymorphism. KIR B haplotypes contain various combinations of both activating and inhibitory KIR genes and show less allelic polymorphism [8, 9].

KIR receptors recognize four mutually exclusive epitopes carried by HLA-A, HLA-B, and HLA-C class I molecules: C1, C2, Bw4, and A3/A11. HLA-C is known to consist of two functional classes of allotypes, depending on an amino acid dimorphism at residue 80 of the α1-helix. The C1 epitope is carried by HLA-C allotypes with asparagine at position 80 and by some HLA-B alleles in the Asian population that have asparagine at position 80 and valine at position 76. The C2 epitope is carried by HLA-C allotypes characterized by a lysine at position 80 of the α1-helix. The Bw4 epitope is carried by HLA-A and HLA-B allotypes that have arginine at position 83. The A3/11 epitope of HLA-A is carried by two HLA-A allotypes, HLA-A*03 and HLA-A*11 [10, 11].

The diversity in gene content of KIR haplotypes and the extensive polymorphism of HLA and KIR genes make it possible for an individual to express KIR genes for which there are no HLA ligands and vice versa, which thus determines extremely variable immunogenetic profiles among individuals [12]. KIR A haplotypes seem to provide better immune surveillance against viral infections and tumor cells, whereas KIR B haplotypes seem to have a favorable role during pregnancy [10].

Although there is a growing interest in the role of NK cells in oncohematologic disorders [13–17], to the best of our knowledge, there is only one previous report investigating the possible involvement of KIR genes in cHL [18]. To gain further insight into the role of NK cells in cHL and their possible impact on response to treatment, we investigated KIR gene frequencies, KIR haplotypes, and KIR-ligand combinations in a cohort of advanced-stage cHL patients compared to healthy controls. Moreover, we evaluated the correlation of KIR genes and KIR haplotypes with the achievement of negative iPET-2 and their possible impact on progression-free survival (PFS).

## Methods

### Ethics, consent, and permissions

In accordance with the 1975 guidelines of the Declaration of Helsinki and after obtaining approval for the study from the four competent local Ethics Committees (Cagliari, Catania, Cuneo, and Reggio Calabria), the medical staff of each participating center exhaustively informed their patients of the potential risks and benefits of the planned treatment and the need for periodical clinical and laboratory checkups.

### Patients, healthy controls, and treatment protocols

One hundred and thirty-five patients with advanced-stage cHL were included in the study. All patients were enrolled in the international prospective multicenter clinical trial HD 0607 (NCT00795613). A complete data set containing all the following parameters was collected at baseline: age, sex, histological subtype, presence of B-symptoms (fever, weight loss exceeding 10 % of body weight in 6 months, night sweats), extranodal involvement or bulky mass, absolute lymphocyte and neutrophil count, hemoglobin and albumin, Ann Arbor stage, and International Prognostic Score (IPS) [19].

All the patients received two courses of standard ABVD treatment before undergoing iPET-2 to assess tumor chemosensitivity. Patients were stratified into two groups according to the results of iPET-2. Patients with positive iPET-2 were randomly assigned to receive four courses of escalated BEACOPP (rituximab, bleomycin, etoposide, doxorubicin, cyclophosphamide, vincristine, procarbazine, prednisone) or R-BEACOPP (BEACOPP supplemented with rituximab), followed by four courses of baseline BEACOPP or R-BEACOPP. Patients with a negative iPET-2 scan continued with four more courses of ABVD. Patients in complete remission with a negative final PET scan who presented bulky disease at baseline or showed a PET-negative residual mass at the end of chemotherapy were randomly assigned to receive consolidation radiotherapy or no further treatment.

A group of 221 healthy subjects (104 males and 117 females) with a mean age of 35 (range 25–55 years) were selected for control purposes.

### HLA and KIR typing

HLA typing was performed in healthy controls and patients using commercially available PCR kits with sequence-specific primers (HLA-A-B-C SSP Combi Tray, Olerup, Stockholm, Sweden, and Micro SSP™ Generic HLA class I typing tray SSP1L, One Lambda, Inc., Canoga Park, CA, USA). HLA-C groups C1 and C2 and HLA-Bw4 were determined by high-resolution typing for the alleles at the HLA-A, HLA-B, and HLA-C loci using a polymerase chain reaction-sequence-specific primer (PCR-SSP) method according to the manufacturers’ instructions (allele-specific PCR-SSP kits: Olerup SSP AB, Stockholm, Sweden).

Distribution of KIR genes (*KIR2DL1*, *KIR2DL2*, *KIR2DL3*, *KIR2DL4*, *KIR2DL5A*, *KIR2DL5B*, *KIR2DS1*, *KIR2DS2*, *KIR2DS3*, *KIR2DS4*, *KIR2DS5*, *KIR3DL1*, *KIR3DL2*, *KIR3DL3*, *KIR3DS1*) were determined by PCR-SSP using a previously reported method [20, 21].

Patients were stratified into two groups according to homozygosity for KIR A haplotype (KIR genotype AA) and heterozygosity or homozygosity for KIR B haplotype (KIR genotypes AB and BB, referred together as KIR genotype Bx) [21, 22].

### Statistical analysis

KIR gene frequencies, KIR haplotypes, and KIR-ligand combinations were compared between cHL patients and healthy controls as well as between cHL patients who achieved negative iPET-2 and those who did not. The abovementioned set of independent variables was included in univariate and multivariate analyses. Where appropriate, significant differences were calculated using Fisher’s two-sided exact test or Pearson’s chi-squared test. Variables with a *p* value lower than 0.2 in univariate analysis were included in multivariate analysis using a multi-step forward binary logistic regression model, where negative iPET-2 scan was considered a dependent variable. Only *p* values ≤0.05 were considered to be statistically significant. The probability of achieving overall survival (OS) and PFS was calculated using the Kaplan-Meier method. Comparisons were also made to assess PFS in patient subgroups according to KIR haplotype or iPET results. The log-rank test was used to compare the groups of patients.

## Results

### Characteristics of patients and controls

The demographic and clinical characteristics of 135 advanced-stage cHL patients (67 males and 68 females, mean follow-up 35 months, range 8–60) and 221 controls are shown in Table 1. The overall mean age at diagnosis was 34 years (range 15–69). The large majority of patients (80 %) had a nodular sclerosing histological subtype. Bulky mass and “B” symptoms were found in 40.7 and 54.1 %, respectively. According to the HD 0607 inclusion criteria, all patients belonged to advanced Ann Arbor stages (IIb–IV) with an unfavorable IPS in 28.9 % of cases. The demographic characteristics of the 221 healthy controls (104 males and 117 females) with a mean age of 35 (range 25–55 years) were not statistically different from the cHL cohort.

**Table 1:** Characteristics of 135 patients with advanced-stage classic Hodgkin lymphoma and 221 healthy controls

	Patients *n* = 135		Controls *n* = 221	*p* value
Sex, no. (%)				
Male	67	(49.6)	104 (47.1)	NS
Female	68	(50.4)	117 (52.9)	NS
Age at diagnosis, mean (range)	34	(15–69)	35 (25–55)	NS
Disease subtype, no. (%)				
Nodular sclerosing	108	(80.0)		
Mixed-cellularity	14	(10.4)		
Lymphocyte-rich	9	(6.7)		
Lymphocyte-depleted	4	(2.9)		
Albumin, g/dl, mean (range)	3.8	(2.2–5.1)		
Hemoglobin, g/dl, mean (range)	13.0	(8.3–14.2)		
Lymphocytes, ×10^3^/μL, mean (range)	1.75	(0.3–4.9)		
Neutrophils, ×10^3^/μL, mean (range)	8.55	(2.1–35.8)		
Bulky mass, no. (%)	55	(40.7)		
“B” symptoms, no. (%)	73	(54.1)		
Extranodal extension, no. (%)	42	(31.1)		
IPS score ≥ 4 (unfavorable in advanced stages)	39	(28.9)		
Ann Arbor stage 1–2a, no. (%)	0	(0)		
Stage 2b, no. (%)	57	(42.2)		
Stage 3, no. (%)	45	(33.3)		
Stage 4, no. (%)	33	(24.5)		

*NS* not significant

### KIR gene frequencies, KIR haplotypes, and KIR-HLA ligand combinations in cHL patients and controls

Table 2 shows KIR gene frequencies, KIR haplotypes, and KIR-HLA ligand combinations in 135 cHL patients and 221 healthy controls. No significant differences were found between patients and controls for the frequencies of the single activating and inhibitory KIR genes. Overall, the healthy controls had significantly higher frequencies of the HLA-C1 ligand (C1/C1 or C1/C2) compared to patients (72.7 vs. 60 %, *p* = 0.014); on the other hand, frequency of the HLA-C2 ligand (C2/C2 or C1/C2) was significantly higher in cHL patients (88.9 vs. 76 %, *p* = 0.003). No significant differences were found for frequencies of the HLA-Bw4 ligand within the two groups. Homozygosity for KIR A haplotype combined with homozygosity for the HLA-C1 ligand (KIRAA/C1C1) was significantly higher in healthy controls (15.7 vs. 4.8 %, *p* = 0.001). The analysis of the combinations of KIR genes and their specific ligands showed a significantly higher frequency of KIR3DS1 and KIR3DL1 when combined with the absence of their specific ligand HLA-Bw4 in cHL patients (15.5 vs. 6.3 %, *p* = 0.006 and 33.3 vs. 21.3 %, *p* = 0.013, respectively).

**Table 2:** KIR and HLA gene frequencies, KIR haplotypes, and KIR-HLA ligand combinations in 135 advanced-stage classic HL patients and 221 healthy controls

	Patients (135)	Percent	Controls (221)	Percent	*p*	OR (95 % CI)
Activating KIR						
KIR2DS1	72	53.3	97	43.9.		
KIR2DS2	78	57.8	125	56.6		
KIR2DS3	54	40.0	77	34.9		
KIR2DS4	124	91.9	202	91.4		
KIR2DS5	42	31.1	75	33.9		
KIR3DS1	53	39.3	88	39.8		
Inhibitory KIR						
KIR2DL1	132	97.8	215	97.2		
KIR2DL2	80	59.2	126	57.0		
KIR2DL3	123	91.1	188	85.0		
KIR2DL4	135	100	221	100		
KIR2DL5	78	57.8	116	52.5		
KIR3DL1	135	100	206	93.2		
KIR3DL2	135	100	219	99.1		
KIR3DL3	135	100	221	100		
KIR genotypes						
AA	42	31.1	66	29.9		
Bx	93	68.8	155	70.1		
AA/C1-C1	2	4.8	35	15.7	0.001	0.08 (0.013–0.35)
KIR ligands						
C1 pres	81	60.0	161	72.7	0.014	0.56 (0.35–0.90)
C2 pres	120	88.9	168	76	0.003	2.5 (1.31–4.90)
HLA-Bw4 pres	90	66.9	175	79.2		
Activating KIR and their ligands						
KIR2DS1 pres/HLA-C2 pres	56	41.5	78	35.3		
KIR2DS1 pres/HLA-C2 abs	16	11.9	19	11.6		
KIR2DS2 pres/HLA-C1 pres	46	34.1	80	36.2		
KIR2DS2 pres/HLA-C1 abs	32	23.7	45	20.4		
KIR3DS1 pres/HLA-Bw4 pres	32	23.7	74	33.5		
KIR3DS1 pres/HLA-Bw4 abs	21	15.5	14	6.3	0.006	2.7 (1.27–5.9)
Inhibitory KIR and their ligands						
KIR2DL1 pres/HLA-C2 pres	100	74.1	164	74.2		
KIR2DL1 pres/HLA-C2 abs	32	23.7	51	23.1		
KIR2DL2 pres/HLA-C1 pres	47	34.8	83	37.6		
KIR2DL2 pres/HLA-C1 abs	32	23.7	43	19.5		
KIR2DL3 pres/HLA-C1 pres	78	57.8	132	59.7		
KIR2DL3 pres/HLA-C1 abs	45	33.3	56	25.3		
KIR3DL1 pres/HLA-Bw4 pres	90	63.7	159	71.9		
KIR3DL1 pres/HLA-Bw4 abs	45	33.3	47	21.3	0.013	1.9 (1.1–3.1)

*abs* absent, *pres* present, *OR* odds ratio, *CI* confidence interval, *Ile* isoleucine, *Thr* threonine

### KIR gene frequencies, KIR haplotypes, and KIR-HLA ligand combinations in cHL patients with positive or negative iPET-2 results

Table 3 shows HLA and KIR gene frequencies, KIR haplotypes, and the combinations between KIRs and their ligands in 33 patients who showed positive iPET-2 in comparison with 102 patients with negative iPET-2.

**Table 3:** KIR and HLA gene frequencies, KIR haplotypes, and KIR-HLA ligand combinations in 135 advanced-stage classic Hodgkin lymphoma patients according to positive or negative interim PET-2 results

	PET-2 positive no. 33	% 24.4	PET-2 negative no. 102	% 75.6	*p*	OR (95 % CI)
Activating KIR						
KIR2DS1	23	69.7	49	48.0		
KIR2DS2	27	81.8	51	50.0	0.001	4.5 (1.6–13.3)
KIR2DS3	16	48.5	38	37.3		
KIR2DS4	28	84.9	96	94.1		
KIR2DS5	9	27.3	33	32.3		
KIR3DS1	16	48.5	37	36.3		
Inhibitory KIR						
KIR2DL1	33	100	99	97.1		
KIR2DL2	22	66.7	58	56.9		
KIR2DL3	30	90.9	93	91.2		
KIR2DL4	33	100	102	100		
KIR2DL5	20	60.6	58	56.9		
KIR3DL1	33	100	102	100		
KIR3DL2	33	100	102	100		
KIR3DL3	33	100	102	100		
KIR genotypes						
AA	5	15.2	37	36.3	0.03	0.31 (0.1–0.95)
Bx	28	84.8	65	63.7		
KIR ligands						
C1 pres	22	66.7	74	72.5		
C2 pres	27	81.8	88	86.3		
HLA-Bw4 pres	24	72.7	66	64.7		
Activating KIR and their ligands						
KIR2DS1 pres/HLA-C2 pres	22	66.7	36	35.3	0.03	7.94 (0.496–173)
KIR2DS1 pres/HLA-C2 abs	1	3.3	13	12.7		
KIR2DS2 pres/HLA-C1 pres	21	63.3	21	20.6	0.004	5.0 (1.55–16.8)
KIR2DS2 pres/HLA-C1 abs	6	18.2	30	29.4		
KIR3DS1 pres/HLA-Bw4 pres	11	33.3	17	16.7		
KIR3DS1 pres/HLA-Bw4 abs	5	15.6	20	19.6		
Inhibitory KIR and their ligands						
KIR2DL1 pres/HLA-C2 pres	27	81.8	59	57.8	0.021	3.05 (1.07–9.11)
KIR2DL1 pres/HLA-C2 abs	6	18.2	40	42.2		
KIR2DL2 pres/HLA-C1 pres	19	62.7	33	32.3	0.018	4.8 (1.2–23.1)
KIR2DL2 pres/HLA-C1 abs	3	9.1	25	24.5		
KIR2DL3 pres/HLA-C1 pres	23	69.7	55	53.9		
KIR2DL3 pres/HLA-C1 abs	7	21.2	38	37.3		
KIR3DL1 pres/HLA-Bw4 pres	22	66.7	65	63.7		
KIR3DL1 pres/HLA-Bw4 abs	11	33.3	37	36.3		

*abs* absent, *pres* present, *OR* odds ratio, *CI* confidence interval, *Ile* isoleucine, *Thr* threonine

A significantly higher frequency of the *KIR2DS2* activating KIR gene was found in patients with positive iPET-2 (81.8 vs. 50 %, *p* = 0.001). Homozygosity for KIR A haplotype was present with a significantly higher frequency in patients with negative iPET-2 (36.3 vs. 15.2 %; *p* = 0.03). In particular, only 5 of the 33 patients with positive iPET-2 had KIR genotype AA, whereas the remaining 28 (84.8 %) had KIR genotype Bx. Homozygosity for KIR A haplotype remained the only predictive factor for the achievement of negative iPET-2 (*p* = 0.039). The analysis of HLA-KIR combinations showed that cHL patients with a positive iPET-2 scan had higher frequencies of the activating KIR genes, *KIR2DS1* and *KIR2DS2*, combined with the presence of their specific ligands C2 and C1, respectively (*KIR2DS1*/HLA-C2: 66.7 vs. 35.3 %, *p* = 0.03; *KIR2DS2*/HLA-C1: 63.3 vs. 20.6 %, *p* = 0.004). Higher frequencies were also observed in this group of patients for the inhibitory KIR genes, *KIR2DL1* and *KIR2DL2*, combined with the presence of their specific ligands C2 and C1, respectively (*KIR2DL1*/HLA-C2: 81.8 vs. 57.8 %, *p* = 0.021; *KIR2DL2*/HLA-C1: 62.7 vs. 32.3 %, *p* = 0.018).

### Overall survival and progression-free survival following treatment and according to KIR haplotype and iPET-2

**Fig. 1:**
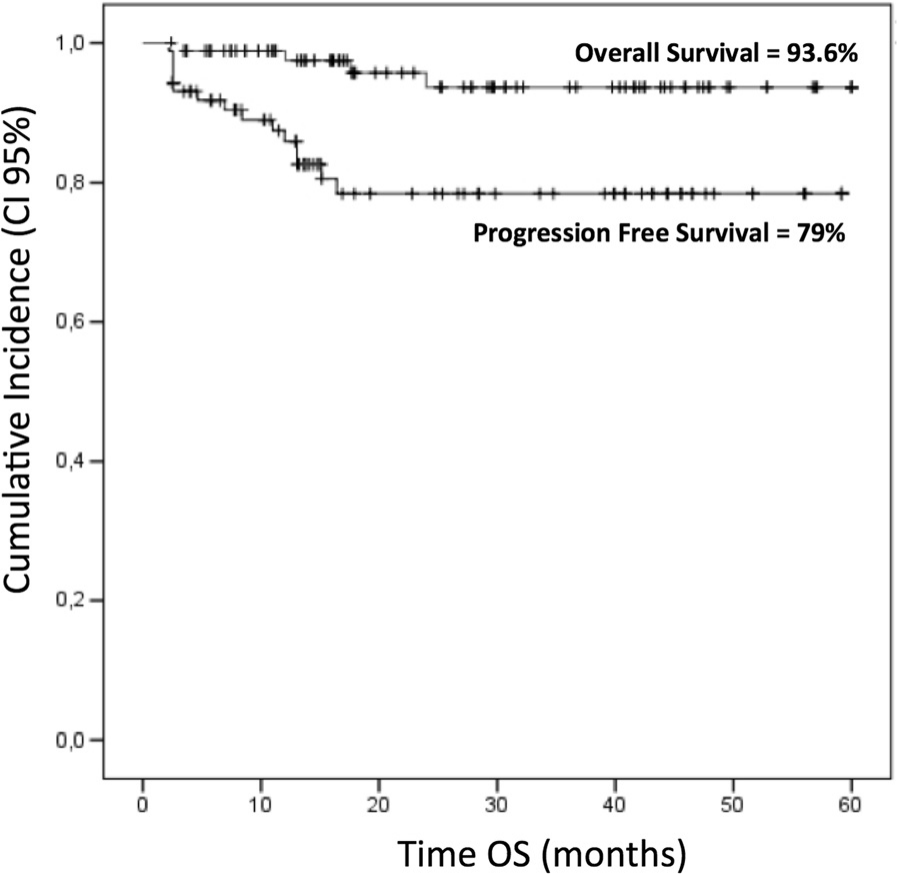
Overall survival (OS) and progression-free survival (PFS) in the cohort of 135 patients. *CI* confidence interval

**Fig. 2:**
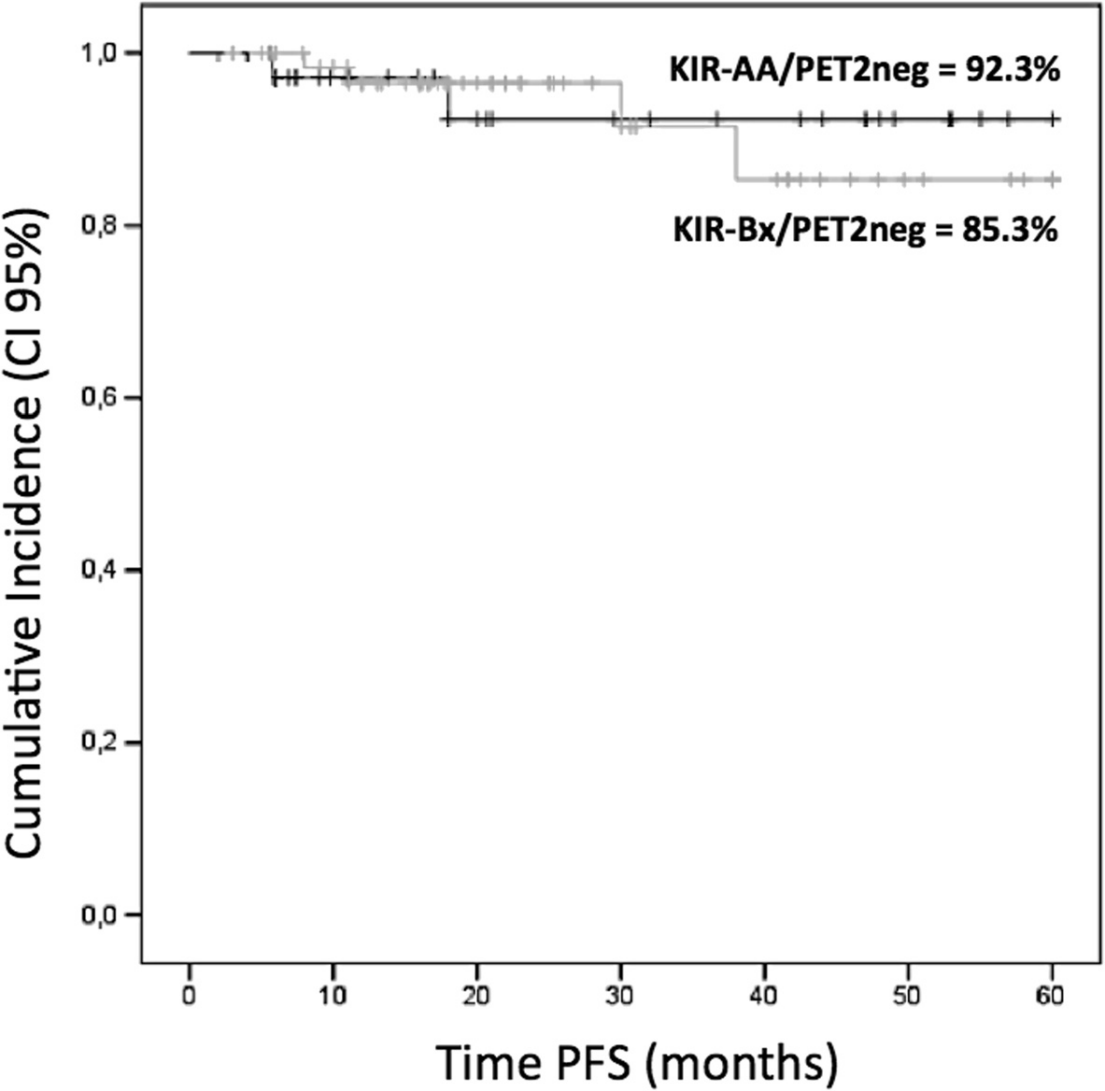
Progression-free survival (PFS) in the cohort of 102 patients with negative interim PET, stratified in two groups according to KIR haplotype (*p* = NS). *CI* confidence interval

In the cohort of 135 patients, 5-year OS and PFS was 93.6 and 79 %, respectively (Fig. 1). PFS was significantly higher in patients who achieved negative iPET compared to those who did not (89.8 vs. 55.8 %, *p* = 0.0001); among patients with negative iPET, those homozygous for KIR A haplotype had higher PFS rates compared to those carrying Bx haplotypes, although the difference was not statistically significant (92.3 vs. 85.3 %) (Fig. 2).

## Discussion

Survival for advanced-stage cHL patients has increased to 70–85 % over the past years, but the optimal balance of the risks and benefits of treatment is still a matter of debate. At 15 years from the end of treatment, the risk of dying from lymphoma is less than the risk of dying from therapy-related complications [23]. The main dilemma remains whether frontline intensive chemotherapy should be given to all patients or be restricted to patients who are poor responders to less intensive treatment approaches. So far, iPET-2 scan represents the only strong predictor of response to treatment [2]. There are currently no other available prognostic biomarkers capable of distinguishing patients requiring more or less intensive treatment. For this reason, many ongoing studies focus their efforts on the definition of new predictive factors for prognosis and response to treatment in cHL. Ideally, risk assessment should combine innovative predictive biomarkers with new imaging technologies [24–27].

Classic HL represents an extremely interesting study model for the assessment of immunologic and immunogenetic factors that may confer susceptibility to tumors or facilitate tumor immune escape mechanisms [4, 5]. Currently few data exist on the role of impaired NK cell function in immune escape of HRS cells.

Besson et al. investigated association between KIRs and cHL in a family study [18]. They provide evidence that activating KIR genes, in particular *KIR2DS1* and *KIR3DS1*, may exert protection in cHL. In our cohort of patients, we found a significantly higher frequency for *KIR3DS1* in the absence of its specific Bw4 ligand, suggesting that this activating KIR gene cannot properly exert its protective function. No other significant differences were observed between cHL patients and controls for single KIR gene frequencies. Vice versa, when we analyzed C1 and C2 epitopes, we found that C2 had a significantly higher frequency in cHL patients (88.9 vs. 76 %, *p* = 0.008). Conversely, the C1 epitope was significantly higher in healthy controls (72.7 vs. 60, *p* = 0.036) (Table 2). Interestingly, homozygosity for KIR A haplotype and HLA-C1 ligand (KIRAA/C1C1) was significantly higher in healthy controls, suggesting a possible protective role in cHL (15.7 vs. 4.8 %, *p* = 0.001) (Table 2).

In a recent review, Parham and Moffett suggest that homozygosity for HLA-C1 and KIR haplotype A could be associated to a better immune response with resistance to viral infection [10]. It is possible that the fixed gene content of KIR A haplotypes with their highly polymorphic receptors provides better immune surveillance against viral infections, whereas KIR B haplotypes with their greater variation in gene content and moderate allelic polymorphism could have an important role in immune-tolerance during pregnancy [10]. Our data seem to suggest a protective role for the KIRAA/C1C1 genotype in cHL.

In our study, KIR A haplotype was the only factor significantly associated to a negative iPET-2 result suggesting its favorable role in the achievement of better response to standard chemotherapy. Moreover, the combination of negative iPET-2 with homozygosity for KIR A haplotype seems to represent a favorable combination for identifying patients responsive to standard courses of chemotherapy (PFS = 92.3 %) (Fig. 2).

Finally, we found that cHL patients with iPET-2 positivity had higher frequencies of the KIR-ligand combinations *KIR2DS1*/HLA-C2 and *KIR3DS2*/HLA-C1. The interaction between *KIR2DS1*, *KIR2DS2*, and their HLA-C ligands might increase the production of tumor growth factor (TGF)-β by NK cells. It has been suggested that TGF-β may reduce tumor lysis mediated by NK cells and stimulate the production of proinflammatory cytokines such as tumor necrosis factor alpha (TNF-α) and interferon gamma (IFN-γ) [28].

## Conclusions

Our results, though preliminary, seem to show that homozygosity for KIR A haplotype, alone or in combination with homozygosity for the HLA-C1 KIR ligand, confers protection to cHL. The association found for the KIR-AA genotype and achievement of negative iPET-2 suggests that testing for KIR haplotypes could be used in clinical practice to enhance the power of iPET-2 in predicting better treatment outcome in cHL patients. Overall, our findings may represent a rationale for developing new risk-adapted treatment strategies based on the combination of KIR biomarkers and PET scans. They furthermore strengthen the hypothesis of innovative and targeted therapies aimed at modulating NK cell function. The use of anti-inhibitory-KIR antibodies could prove to be an effective way of blocking the binding interface of inhibitory KIRs with HLA class I ligands expressed by lymphoma cells, thus preventing a tolerogenic interaction and augmenting NK cell cytotoxicity [28–31].
